# Lipin-1 Drives Browning of White Adipocytes via Promotion of Brown Phenotype Markers

**DOI:** 10.3390/biomedicines13092069

**Published:** 2025-08-25

**Authors:** Siti Sarah Hamzah, Liyana Ahmad Zamri, Siti Azrinnah Abdul Azar, Siti Mastura Abdul Aziz, Shazana Rifham Abdullah, Norhashimah Abu Seman

**Affiliations:** Endocrine and Metabolic Unit, Nutrition, Metabolic & Cardiovascular Research Centre, Institute for Medical Research, National Institutes of Health, Ministry of Health Malaysia, Setia Alam 40170, Selangor, Malaysia; liyana.az@moh.gov.my (L.A.Z.); azrinnah@moh.gov.my (S.A.A.A.); sitimastura.aziz@moh.gov.my (S.M.A.A.); shazana.a@moh.gov.my (S.R.A.); nor.hashimah@moh.gov.my (N.A.S.)

**Keywords:** lipin-1, SIRT1, adipocytes, beige/brown, obesity

## Abstract

**Background:** Enhancing adipose tissue functionality is a promising cellular-level approach to combating obesity. White adipose tissue (WAT) can acquire beige or brown adipose tissue (BAT)-like properties, characterized by increased thermogenesis and energy dissipation. While the SIRT1-SRSF10–Lipin-1 axis has been identified in hepatocytes, where Lipin-1 regulates triglyceride metabolism, its role in adipocytes remains unclear. This study aimed to investigate the function of Lipin-1 in 3T3-L1 preadipocytes and its interaction with SIRT1, SRSF10, and PPARγ in promoting browning-like transcriptional responses. **Methods:** Mouse 3T3-L1 preadipocytes were treated during differentiation with either rosiglitazone (RGZ), the SIRT1 activator SRT1720, or the SIRT1 inhibitor EX527. Gene expression was assessed by real-time PCR, and protein levels were measured using the Simple Western blot system. Data were compared with untreated controls and analyzed using GraphPad Prism. **Results:** Lipin-1 expression was significantly upregulated by RGZ treatment, alongside increased transcription of Sirt1 and Srsf10, supporting the presence of this regulatory axis in adipocytes. Elevated Srsf10 favored the production of the Lipin-1b isoform, whereas SIRT1 inhibition reversed these effects, confirming its upstream role. Pathway activation further enhanced the expression of browning markers, including Ucp1, Pgc1a, PRDM16, and CIDEA. **Conclusions:** These findings demonstrate that Lipin-1 interacts with the SIRT1–PPARγ–SRSF10 axis in adipocytes and contributes to the acquisition of beige/brown-like characteristics in WAT. This regulatory pathway may represent a potential target for improving lipid metabolism and metabolic health.

## 1. Introduction

Obesity is a global health crisis characterized by excessive body fat accumulation, significantly increasing the risk of chronic conditions such as type 2 diabetes, cardiovascular disease, and certain cancers. At the cellular level, obesity is associated with complex alterations in adipose tissue, where adipocytes play a central regulatory role [1,2]. Enhancing adipocyte function presents a promising strategy for mitigating obesity and its related complications. Healthy adipocytes are essential for maintaining metabolic balance by regulating energy storage and release, secreting adipokines, and preserving systemic homeostasis [3,4]. In contrast, dysfunctional adipocytes contribute to insulin resistance, chronic inflammation, and the progression of obesity-related disorders [5,6].

Recent research has increasingly focused on strategies to improve adipocyte functionality, with particular emphasis on the browning capability of white adipocytes. Browning refers to the process by which white adipocytes (WAT) acquire characteristics of beige/brown adipocytes (BAT), including enhanced thermogenic capacity [7,8,9]. This process is associated with increased calorie burning and improved metabolic health. A key regulator of this process is SIRT1 (Sirtuin 1), a sirtuin family deacetylase that modulates multiple metabolic pathways and cellular stress responses [10,11,12,13]. SIRT1 promotes browning by regulating the expression of thermogenic and lipid metabolism-related genes.

Lipid metabolism within adipocytes is also tightly regulated by Lipin-1 (*Lpin1*), a phosphatidic acid phosphatase crucial for both lipid synthesis and breakdown [14,15,16,17]. Studies in fatty liver dystrophy (fld) mice have demonstrated that Lipin-1 is responsible for all phosphatidic acid phosphatase (PAP) activity in adipose tissue and skeletal muscle, where it is the predominant lipin isoform [18,19]. In contrast, Lipin-1 contributes only partially to PAP activity in the liver, heart, kidney, and brain [20,21,22]. Therefore, investigating the pathways regulated by SIRT1 and Lipin-1 in adipocytes may offer new avenues for improving adipocyte function.

Interestingly, SRSF10, a splicing factor involved in the alternative splicing of Lipin-1 pre-mRNAs, has emerged as a potential molecular link between SIRT1 and Lipin-1 signaling [23,24,25,26]. Each component—SIRT1, SRSF10, and Lipin-1—has been independently implicated in lipid metabolism and the pathogenesis of fatty liver disease [24]. Given that the transcription factor PPARγ is known to enhance Lipin-1 expression [27,28], we used the PPARγ agonist, rosiglitazone (RGZ), to stimulate Lipin-1expression in mouse 3T3-L1 cells. While the individual roles of SIRT1, SRSF10, and Lipin-1 in metabolic regulation are well documented, their potential interactions within adipocytes and their contribution to the acquisition of beige/brown characteristics by white adipocytes remain poorly understood. To address this gap, we investigated the regulatory interactions between Lipin-1 and SIRT1, PPARγ, and SRSF10 during adipogenesis in mouse 3T3-L1 cells. Additionally, we aimed to determine whether these interactions play a potential role in promoting the transcription of genes involved in the browning of white adipocytes in vitro.

## 2. Materials and Methods

### 2.1. Cell Culture

First, 3T3-L1 cells (Elabscience, Cat. No: EP-CL-0006, Houston, TX, USA) were cultured in DMEM (Nacalai Tesque, Product No: 08460-95, Kyoto, Japan), supplemented with 10% fetal bovine serum (FBS) (Tico Europe, Product No: FBSEU500, Amstelveen, The Netherlands) and 1% penicillin–streptomycin (P/S) (Nacalai Tesque, Product No: 09367-34), at 37 °C in a humidified incubator with 5% CO_2_. Adipocyte differentiation was induced using the protocol described in [29], with some modifications. Confluent cells were maintained for an additional two days in induction medium consisting of DMEM with 10% (*v*/*v*) FBS, 0.5 mM IBMX (Nacalai Tesque, CAS No: 28822-58-4), 1 µM dexamethasone (DEX) (Nacalai Tesque, CAS No: 50-02-2), and 10 µg/mL insulin (INS) (Nacalai Tesque, CAS No: 11061-68-0). After this induction period, the medium was replaced every other day for an additional eight days with DMEM containing 10% (*v*/*v*) FBS and 10 µg/mL INS, supplemented with one of the following compounds: rosiglitazone (RGZ) (5 μM) (Tokyo Chemical Inc., CAS. No: 122320-73-4, Tokyo, Japan), the SIRT1 activator SRT 1720 (2.5 μM) (Santa Cruz Biotechnology, Product. No: SC-364624, Dallas, TX, USA), or the SIRT1 inhibitor EX-527 (10 μM) (ChemFaces, CAS. No: 49843-98-3, Wuhan, China). These treatments were administered continuously throughout the 8-day differentiation period. Parallel control cells were maintained under identical conditions but without any of the three compounds.

A cell viability assay (MTT assay) [30] was performed to identify the optimal concentration of SRT1720, with 2.5 μM selected because of its minimal impact on cell viability throughout the differentiation period. EX-527 was used at a concentration of 10 μM, consistent with previous studies employing this dose to inhibit SIRT1 enzymatic activity [31] and potentially reduce its mRNA expression. Although EX-527 primarily functions by inhibiting SIRT1 activity, prolonged exposure may lead to transcriptional downregulation of SIRT1 by the end of the differentiation period, potentially via feedback mechanisms involving downstream transcription factors.

Adipocyte differentiation was evaluated by microscopic observation of intracellular lipid droplet formation.

### 2.2. Protein Analysis

Total protein was extracted from the cells using a commercial protein extraction kit (Thermo Fisher, Waltham, MA, USA), following the manufacturer’s instructions. Western blotting was performed using the Simple Western Jess Technology, which integrates capillary electrophoresis with conventional Western blotting principles [32]. This system employs Total Protein Normalization (TPN), in which all proteins in the sample are covalently immobilized and detected using a fluorescent total protein stain. The resulting signal reflects the total protein content loaded into each capillary, serving as an accurate internal loading control. As a result, normalization to housekeeping proteins is no longer required. Protein detection was carried out using primary antibodies against Lipin-1 (Novus Biologicals, Centennial, CO, USA; Cat. No: NB110-57150), UCP1 (R&D Systems, Minneapolis, MN, USA; Cat. No: MAB-6158), PGC1 alpha (Novus Biologicals, Centennial, CO, USA; Cat. No: NBP1-04676), and PPAR alpha (Novus Biologicals, Centennial, CO, USA; Cat. No: NB600-636). Protein signals were visualized using the Jess system’s integrated imaging platform. Quantitative analysis was conducted using the system’s software, which measures the intensity of each target protein band and normalizes it to the corresponding total protein signal for each sample.

### 2.3. Gene Expression Analysis

Total RNA was isolated from the 3T3-L1 cells using the RNeasy Mini Kit (Qiagen, Hilden, Germany). For cDNA synthesis, 200 ng of total RNA was reverse-transcribed using ReverTRa Ace™ qPCR RT Master Mix with gDNA remover (Toyobo Co. Ltd., Osaka, Japan). Quantitative real-time PCR was performed using THUNDERBIRD™ Next SYBR^®^ qPCR Mix (Toyobo Co., Ltd., Osaka, Japan) on a StepOnePlus™ RealTime PCR System (Thermo Fisher, Waltham, MA, USA). Relative mRNA expression levels were calculated using the ∆∆Ct method, with b-Actin serving as the internal control. The primer sequences (Integrated DNA Technologies, ICT, Coralville, IA, USA) used in this study are listed in Table 1. These sequences were adopted from the previously published literature, where they were validated for specificity and efficacy in targeting the respective genes [33].

### 2.4. Determination of Lipid Accumulation by Oil Red O Staining

At the end of the differentiation process, the cells were washed twice with PBS (Nacalai Tesque, CAS No: 11482-15) and fixed in 1 mL of paraformaldehyde (Sigma-Aldrich, CAS No: 30525-89-4, St. Louis, MO, USA) for 30 min at room temperature. Following fixation, the cells were stained with filtered Oil Red O (Nacalai Tesque, Product No: 25633-92) solution for 1 h. Excess stain was then carefully removed, and the plates were rinsed twice with PBS. The stained cells were imaged using an inverted microscope (Nikon Eclipse TS2, Nikon Instruments Inc., Melville, NY, USA). To quantify lipid accumulation, the red-stained lipid droplets were extracted using isopropanol, and the absorbance of the extract was measured at 520 nm.

### 2.5. Statistical Analysis

Statistical analysis was performed using one-way analysis of variance (ANOVA) followed by Tukey’s post hoc test for comparisons involving more than two groups or Student’s *t*-test for comparisons between two independent groups. Data were analyzed using GraphPad Prism 5 (GraphPad Software Inc., San Diego, CA, USA). A *p*-value of < 0.05 was considered statistically significant. All results are presented as the mean ± standard error of the mean (SEM) from three independent biological replicates.

## 3. Results

### 3.1. PPARγ-Driven Regulation of Lipin-1 via the SIRT1–SRSF10 Axis

**Figure 1:**
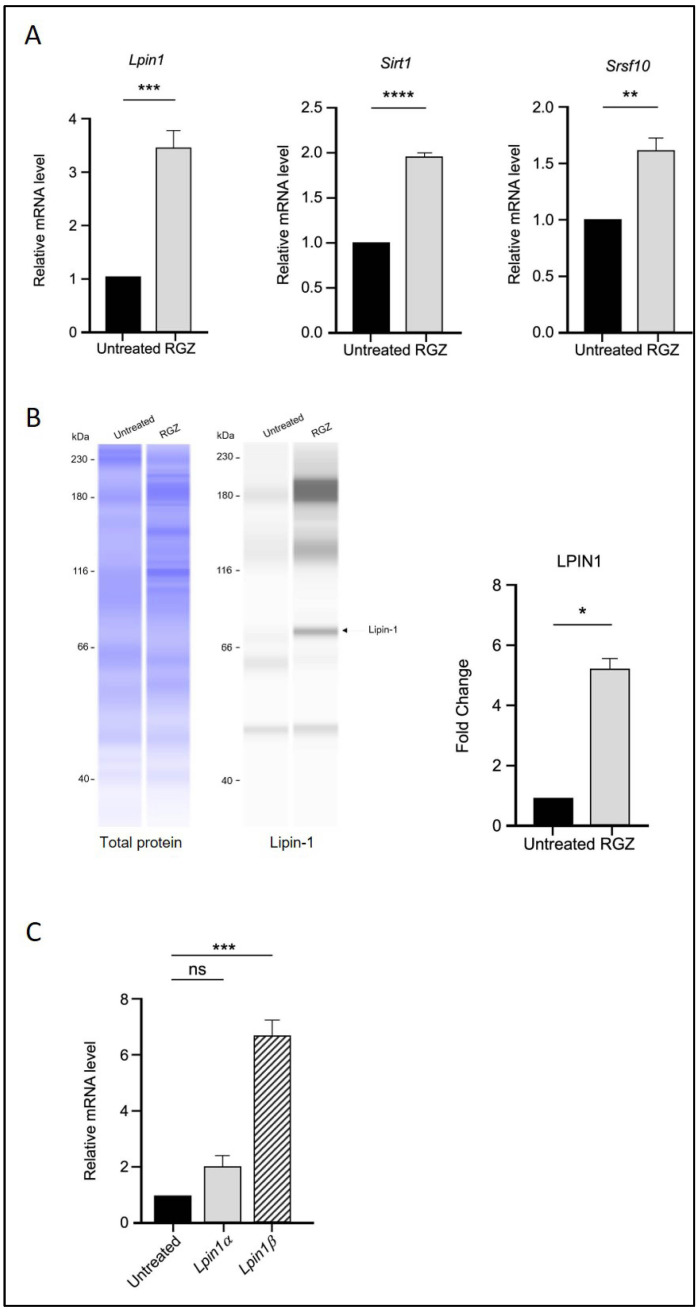
PPARγ activation upregulates Lipin-1, SIRT1, and SRSF10 expression and alters Lipin-1 isoform splicing in 3T3-L1 adipocytes. (**A**) Relative mRNA expression of *Lpin1*, *Sirt1*, and *Srsf10* following PPARγ activation by 5 μM RGZ on Day 8 of differentiation. (**B**) Protein expression of Lipin-1 in RGZ-treated cells compared with untreated controls. (**C**) mRNA expression of *Lpin1a* and *Lpin1b* isoforms in the same treatment group. Statistical significance was assessed by Student’s *t*-test for (**A**,**B**) and one-way ANOVA followed by Tukey’s post hoc test for (**C**), comparing treated groups to the untreated control. Data are presented as means ± SEM from three independent experiments. * *p*  <  0.05; ** *p*  <  0.01; *** *p*  <  0.001; **** *p*  <  0.0001; ns, not significant.

The effect of PPARγ activation by RGZ on *Lpin1* transcription was first examined. As expected, treatment with RGZ resulted in a significant increase in both Lpin1 mRNA and protein levels compared to the untreated group (Figure 1A,B), consistent with previous studies [28].

The transcriptional levels of *Sirt1* and *Srsf10* were then assessed using quantitative real-time PCR following RGZ treatment. An upregulation of *Sirt1* was observed, supporting the involvement of PPARγ in molecular pathways regulated by SIRT1 during adipogenesis. Additionally, the expression level of the splicing factor *Srsf10* was significantly increased in the treated group (Figure 1A). Since Lipin-1 is a splice target of SRSF10, the mRNA expression of its isoforms, *Lpin1a* and *Lpin1b*, was also analysed. The results showed that *Lpin1b* expression was significantly higher than that of *Lpin1a*, suggesting that elevated *Srsf10* levels correlate with *Lpin1* splicing, favoring the production of the *Lpin1b* isoform (Figure 1C).

**Figure 2:**
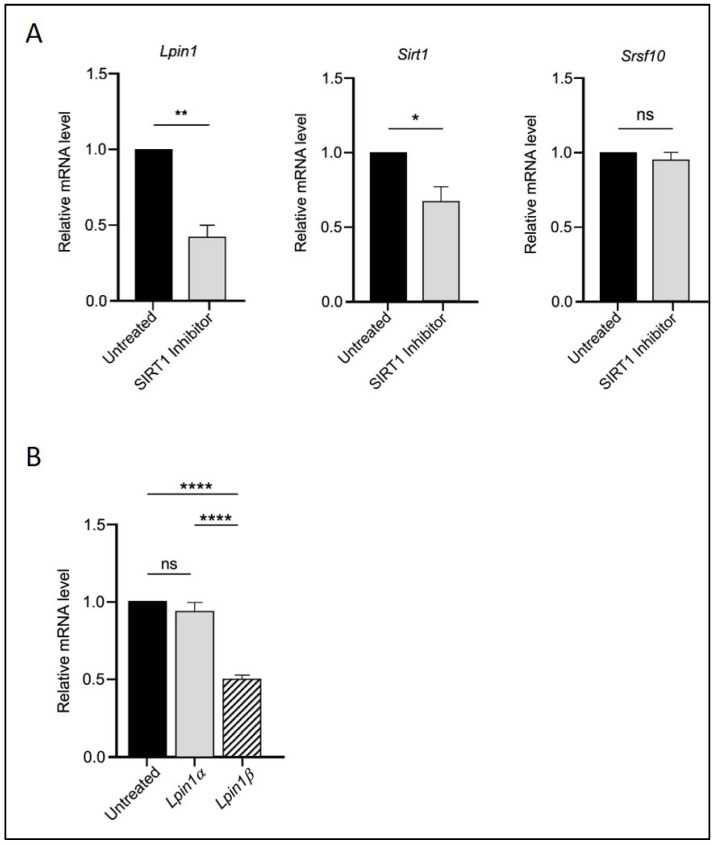
SIRT1 acts upstream in the regulatory interaction with SRSF10 and Lipin-1. (**A**) Relative mRNA expression of *Lpin1*, *Sirt1*, and *Srsf10* in 3T3-L1 adipocytes treated with 10 μM EX527 (SIRT1 inhibitor). (**B**) Expression of *Lpin1a* and *Lpin1b* isoforms in the same treatment group. Statistical significance was assessed by Student’s *t*-test for (**A**) and one-way ANOVA followed by Tukey’s post hoc test for (**B**), comparing treated groups to the untreated control. Data are presented as means ± SEM from three independent experiments. * *p*  <  0.05; ** *p*  <  0.01; **** *p*  <  0.0001; ns, not significant.

To assess whether SIRT1 acts upstream in this regulatory cascade, we treated the cells with the SIRT1 inhibitor EX527. As shown in Figure 2, EX527 reversed the effects of RGZ on Sirt1, Srsf10, *Lpin1* (Figure 2A), and the *Lpin1a*-to-*Lpin1b* ratio (Figure 2B), confirming the upstream role of SIRT1 in this pathway. Overall, these findings suggest a functional interaction among SIRT1, SRSF10, and Lipin-1 that may positively influence adipogenesis and metabolic regulation in 3T3-L1 cells.

### 3.2. Modulation of Lipid Accumulation by SIRT1, SRSF10, and Lipin-1 During Adipocyte Differentiation

**Figure 3:**
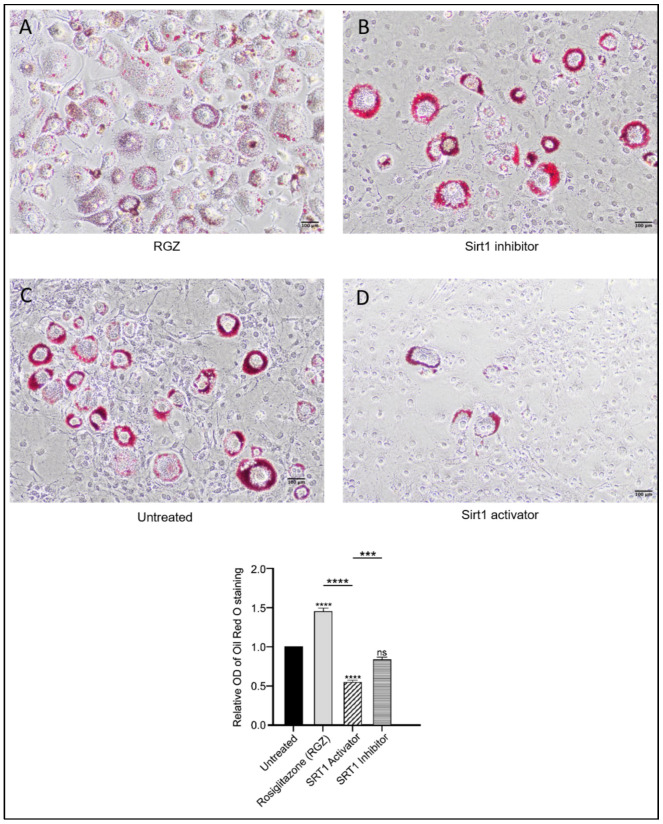
Effects of SIRT1-SRSF10–Lipin-1 upregulation on lipid accumulation in 3T3-L1 preadipocytes. Scale bar: 100 μm. Oil Red O staining of 3T3-L1 cells differentiated for 8 days showed increased and dispersed lipid accumulation with RGZ treatment (**A**). Cells treated with EX-527 (**B**) resembled the untreated group (**C**), displaying localized lipid droplets, whereas SRT1720 treatment (**D**) showed reduced lipid accumulation. Statistical significance was assessed by Student’s *t*-test, comparing treated groups to the untreated control. Data are presented as means ± SEM from three independent experiments. *** *p*  <  0.001; **** *p*  <  0.0001; ns, not significant.

Intracellular lipid droplets were stained with Oil Red O to assess the differentiation of 3T3-L1 preadipocytes following the upregulation of *Lpin1*, *Sirt1*, and *Srsf10*. As shown in Figure 3, lipid accumulation increased in the cells treated with RGZ, with lipid droplets displaying a more dispersed distribution compared to the untreated cells, consistent with previous findings [34]. In contrast, the cells treated with the SIRT1 inhibitor EX527 exhibited more concentrated lipid accumulation in fewer cells, resembling the pattern observed in the untreated group. To explore whether pharmacological activation of SIRT1 might produce effects similar to its indirect activation through PPARγ stimulation, the cells were treated with the SIRT1 activator SRT1720. Treatment with SRT1720 was associated with a significant reduction in lipid accumulation, as further supported by the quantitative absorbance measurements.

### 3.3. Elevated Lipin-1 Expression Enhances Browning Marker Expression via Distinct SIRT1-and PPARγ-Dependent Mechanisms

**Figure 4:**
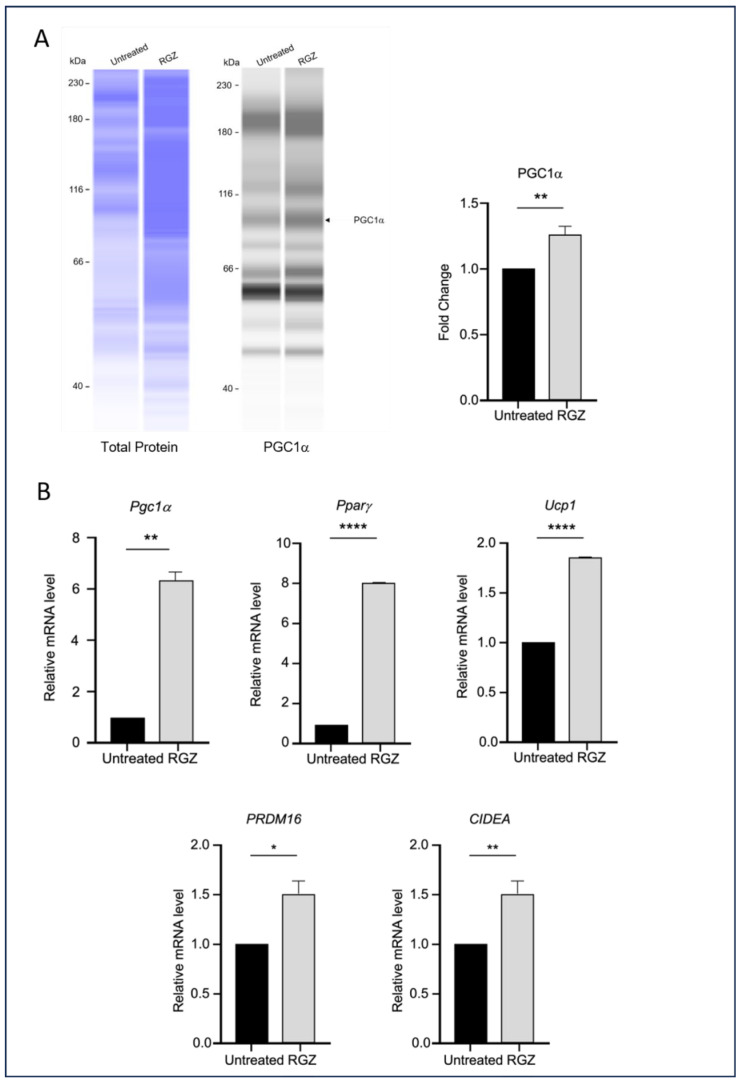
Lipin-1 upregulates brown adipocyte markers. (**A**) PGC1α protein expression and (**B**) mRNA levels of *Pgc1a*, *Pparg*, *Ucp1*, *PRDM16*, and *CIDEA* were increased following Lipin-1 induction with RGZ treatment. Statistical significance was assessed by Student’s *t*-test, comparing treated groups to the untreated control. Data are presented as means ± SEM from three independent experiments. * *p*  <  0.05; ** *p*  <  0.01; **** *p*  <  0.0001.

This study also examined the impact of elevated Lipin-1 expression on the transcription of genes associated with the browning of white adipocytes during differentiation. As shown in Figure 4, the RGZ-treated cells exhibited a significant increase in the expression of key brown adipocyte markers, including PGC1α protein (Figure 4A), and *Pgc1a*, *Pparg*, *Ucp1*, *PRDM16*, and *CIDEA* mRNA (Figure 4B).

**Figure 5:**
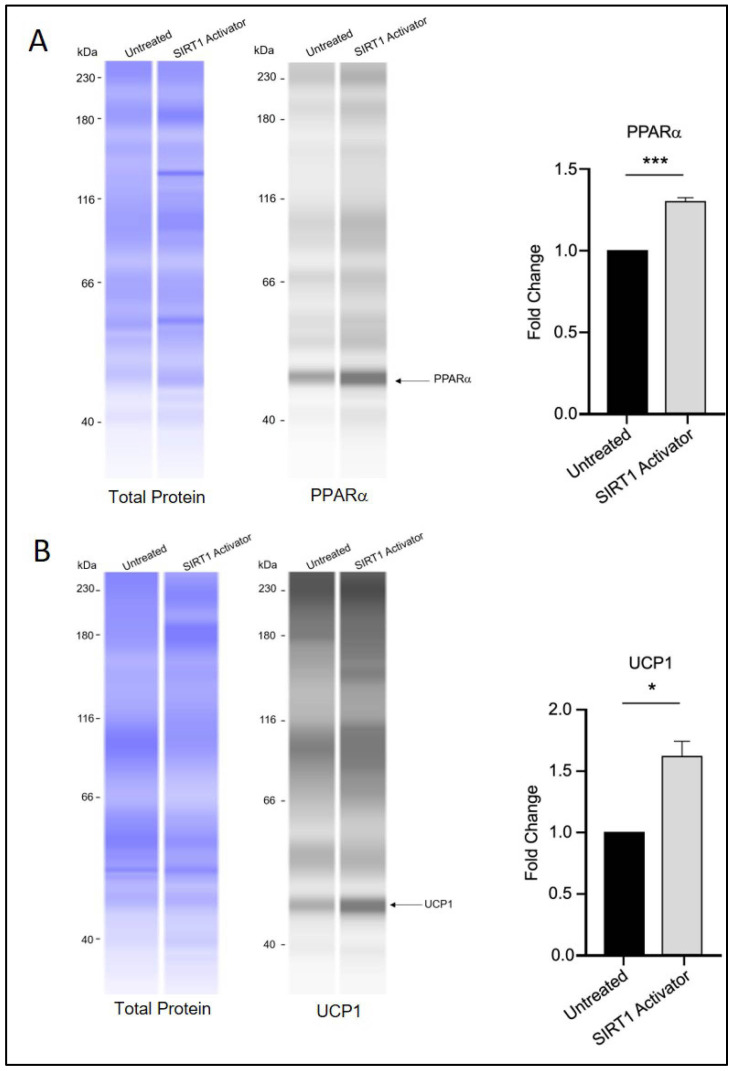
SIRT1 activation and brown adipocyte marker expression. (**A**) PPARα and (**B**) UCP1 protein levels appeared elevated in 3T3-L1 cells treated with SRT1720 during the differentiation period. Statistical significance was assessed by Student’s *t*-test, comparing treated groups to the untreated control. Data are presented as means ± SEM from three independent experiments. * *p * <  0.05; *** *p*  <  0.001.

Interestingly, treatment with SRT1720 was associated with increased protein levels of PPARα and UCP1 (Figure 5A,B), which may indicate a potential role of SIRT1 in influencing the transition of white adipocytes toward a beige/brown phenotype through a mechanism that could differ from that of RGZ treatment. Notably, the UCP1 signal was detected at approximately 47 kDa, which is slightly higher than its commonly reported molecular weight (37 kDa). This discrepancy is likely due to the different separation matrix chemistry used in the Simple Western system, which can result in secondary interaction with the matrix and migration patterns that differ from those seen in conventional Western blotting. Nevertheless, according to the manufacturer’s technical notes, Simple Western and conventional Western blotting generally show good correlation in protein molecular weight estimation. In our results, a distinct fluorescent peak was consistently observed just above 40 kDa across all replicate experiments, clearly standing out from other non-specific signals. Replication data for UCP1 detection are provided in Appendix A.

## 4. Discussion

**Figure 6:**
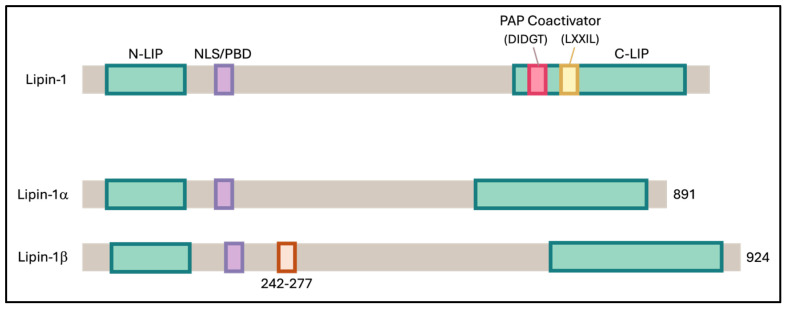
Schematic representation of mouse Lipin-1 and its α and β isoforms. Both isoforms contain two conserved regions: the N-terminal Lipin (NLIP) domain, which is implicated in protein–protein interactions, and the C-terminal Lipin (CLIP) domain, which is important for lipid metabolism. Lipin-1β differs from Lipin-1α by the inclusion of exon 7, introducing an additional 33 amino acids within the linker region between NLIP and CLIP.

Addressing obesity is a complex challenge that demands a comprehensive, multi-level approach to ensure sustainable, safe, and long-term success. One promising strategy is to target obesity at the cellular level, particularly by enhancing the function of adipose tissue. Adipose tissue plays a crucial role in regulating insulin sensitivity by releasing insulin-sensitizing factors such as adiponectin and sequestering lipids, thereby preventing their accumulation in other tissues, which could otherwise result in detrimental effects [35]. Research has shown that white adipose tissue (WAT) can acquire a beige or brown adipose tissue (BAT) phenotype through a process known as browning. These beige adipocytes/BAT are distinct from WAT as they have better thermogenic ability due to higher mitochondrial activity, multilocular lipid droplets, and abundant expression of the uncoupling protein 1 (UCP1) [35]. Lipin-1, an enzyme involved in lipid metabolism, exists in several isoforms, including lipin-1α, lipin-1β, lipin-1γ, lipin-2, and lipin-3 (Figure 6). In adipocytes, Lipin-1 is predominantly found in two main isoforms, lipin-1α and lipin-1β [15]. In hepatocytes, Lipin-1 has been shown to interact with SIRT1 and the splicing factor SRSF10, thereby regulating triglyceride levels and lipogenesis [23]. It also exerts dual functions through PPARγ activation, acting both as a transcriptional coactivator and as a phosphatidate phosphatase, thereby promoting adipocyte differentiation and lipid accumulation [28]. Nevertheless, the interactions between these molecules and their potential roles in adipogenesis and the browning of white adipocytes remain largely unexplored.

In this study, we examined the regulatory interactions within the SIRT1–PPARγ–SRSF10–Lipin-1 axis in 3T3-L1 white preadipocytes. Our results support the presence of this axis, with expression patterns of its components appearing closely correlated. Inhibition of SIRT1 activity was accompanied by reduced *Lpin1* expression, particularly the *b* isoform, which may indicate that SIRT1 plays a positive role in regulating Lipin-1 during adipogenesis. Interestingly, these findings differ from observations in hepatocytes, where Li et al. [24] reported that SIRT1 downregulation decreased *Srsf10* expression but paradoxically increased *Lpin1*, *Lpin1b*, and the *β*:*α* isoform ratio, thereby contributing to metabolic dysregulation. Such discrepancies may reflect cell-type-specific regulatory mechanisms or variations in upstream signaling pathways influencing SIRT1–Lipin-1 interactions. Previous studies have also shown that SIRT1 can deacetylate and modulate PPARγ activity, indicating a bidirectional regulatory relationship that may, in turn, affect *Lpin1* expression and splicing [10,36,37]. In our study, activation of PPARγ with RGZ not only increased *Lpin1* expression but also upregulated *Sirt1*, suggesting a potential feedback loop in which PPARγ activation reinforces SIRT1-mediated transcriptional regulation during adipogenesis.

Chemical inhibition of SIRT1 in our model also led to reduced expression of both *Lpin1* and *Srsf10*, further supporting the role of SIRT1 as an upstream regulator within this regulatory axis. While SIRT1 inhibitors are primarily designed to block its enzymatic activity, it is worth noting that prolonged exposure, such as in the context of this study, may also influence *Sirt1* transcription. These findings suggest that SIRT1 could influence Lipin-1 expression both directly and indirectly, potentially through effects on splicing factors such as SRSF10. Alternative pre-mRNA splicing is known to undergo dynamic remodeling during adipogenesis [38], and SRSF10 has been proposed as a key splicing factor in this process. Although our study did not experimentally confirm a direct role for SRSF10 in Lipin-1 splicing, the parallel expression patterns of *Srsf10* and *Lpin1* observed here are consistent with a molecular network in which SRSF10 may contribute to the regulation of Lipin-1 isoform production, in line with previous reports.

Specifically, SRSF10 has been reported to regulate *Lpin1* splicing in differentiating C3H10T1/2 cells, favoring the production of the Lipin-1a isoform—an effect that promoted adipocyte differentiation more effectively than overexpression of the Lipin-1b isoform in the same model [39]. In contrast, our findings in 3T3-L1 adipocytes showed that higher *Srsf10* expression was associated with increased total *Lpin1*, elevated *Lpin1b* levels, and a greater *Lpin1b*-to-*Lpin1a* ratio. Since the a isoform is more prevalent in undifferentiated preadipocytes and the *b* isoform is enriched in mature adipocytes [21], this isoform shift toward Lipin-1b may reflect progression toward a more differentiated state. Conversely, under conditions of SIRT1 inhibition—where both *Srsf10* and *Lpin1* were downregulated—we observed a relative increase in *Lpin1a* and a higher *Lpin1a*-to-*Lpin1b* ratio. This pattern may indicate a partial maintenance of, or reversion toward, a preadipocyte-like state.

In our study, RGZ treatment was associated with increased lipid accumulation in 3T3-L1 adipocytes, accompanied by improved lipid redistribution. This finding is in line with the observations of Pihlajamäki et al., who reported similar effects in the liver and skeletal muscle of obese humans and high-fat-fed mice [40]. The ability of RGZ to enhance lipid storage during adipocyte differentiation is well documented, largely through upregulation of lipogenic genes via PPARγ activation [41,42]. Consistent with this, we also observed an increase in *Pparg* expression, which may contribute to changes in lipid handling similar to those reported by Li et al. and Sun et al. [43,44]. Together, these results support the role of RGZ in promoting adipocyte maturation and accelerating lipid droplet formation relative to untreated controls.

In contrast, treatment with a SIRT1 activator was associated with reduced lipid storage. Although the precise mechanisms were not examined in our study, this outcome may reflect engagement in alternative molecular pathways linked to SIRT1 activation. Previous studies suggest that pharmacological activation of SIRT1 can influence lipid metabolism by promoting lipolysis or enhancing pathways of lipid catabolism [45,46]. Thus, the divergent effects of RGZ and SIRT1 activator treatments highlight the potential for distinct outcomes when modulating different nodes of the regulatory network governing adipocyte differentiation and lipid metabolism.

**Figure 7:**
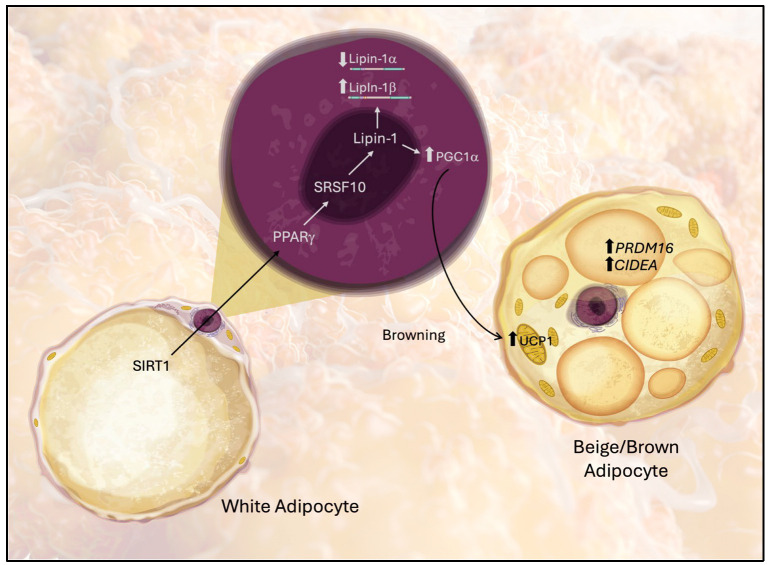
Proposed model of the molecular pathway through which Lipin-1 mediates the acquisition of beige/BAT-like characteristics of white adipose tissue (WAT). Arrows indicate molecular interactions between the pathway components.

Our findings indicate that upregulation of Lipin-1 is associated with increased expression of genes involved in the browning of white adipose tissue (WAT), suggesting a shift toward a brown adipose tissue (BAT)-like phenotype, as illustrated in Figure 7. Browning markers, including *Ucp1*, *Pparg*, *PRDM16, CIDEA*, and *Pgc1a* (a coactivator of mitochondrial biogenesis), were elevated under these conditions. These observations are in line with the work of Wang et al. [47], who demonstrated that knockdown of mTOR or Lipin-1 in 3T3-L1 cells—through both genetic silencing and pharmacological inhibition—reduced the expression of browning-associated genes, such as *Ucp1*, *Pgc1a*, *Cidea*, *Cox7a1*, *Cox8b*, and *Ppra* [48,49]. Key transcriptional regulators such as PRDM16 and lipid droplet-associated proteins like CIDEA are recognized determinants of the brown adipocyte phenotype [50,51], and their regulation by PPARγ has been demonstrated in RGZ-treated models [52]. Moreover, SIRT1 activation in our study was accompanied by elevated PPARα and UCP1, consistent with previous reports that SIRT1 promotes mitochondrial biogenesis and cooperates with PPAR signaling to facilitate browning.

A key strength of this study is that it is among the first to propose the existence of a SIRT1–PPARγ–SRSF10–Lipin-1 axis in adipocytes. Our findings collectively suggest that this pathway contributes to the induction of a thermogenic program in differentiated 3T3-L1 adipocytes, as indicated by elevated transcription of Ucp1 and Pgc1a. Nevertheless, several limitations should be acknowledged. Functional validation, such as measurements of oxygen consumption or mitochondrial respiration, was not performed due to resource constraints and the lack of specialized instrumentation. In addition, we did not conduct splicing assays to directly confirm the role of SRSF10 in regulating Lipin-1 isoform expression. Finally, as these experiments were performed in a mouse cell line, further studies in human adipocytes will be essential to establish the translational relevance of this regulatory axis.

## 5. Conclusions

In summary, this study suggests that SIRT1 may influence PPARγ–Lipin-1 transcriptional activity through SRSF10-dependent splicing, introducing a new regulatory layer in adipocyte phenotype and metabolic function. Given the established role of SIRT1 in mitochondrial biogenesis and the involvement of PPARγ/Lipin-1 in lipid handling, this pathway may contribute to the balance between lipid storage and oxidation, with potential implications for obesity, dyslipidemia, and insulin resistance. While SIRT1 activators (e.g., resveratrol, SRT1720) and PPARγ modulators (e.g., thiazolidinediones) are already available, our findings point to the possibility of developing more selective interventions targeting splicing or Lipin-1 activity to enhance insulin action and glucose homeostasis. However, in vivo validation remains essential, as the physiological outcomes of modulating this axis are likely to be context-dependent. Careful titration of pathway activity will be necessary to maximize metabolic benefits while minimizing adverse effects. Overall, these findings highlight a potential role for the SIRT1–PPARγ–SRSF10–Lipin-1 axis in adipocytes, providing a preliminary framework for future investigations in human cells and in vivo models.

## Figures and Tables

**Table 1:** List of primers.

RT-qPCR Primer	Sequence
*Ucp1* forward	5′-GGC ATT CAG AGG CAA ATC AGC T-3′
*Ucp1* reverse	5′-CAA TGA ACA CTG CCA CAC CTC-3′
*Sirt1* forward	5′-TGC CAT CAT GAA GCC AGA GA-3′
*Sirt1* reverse	5′-AAC ATC GCA GTC TCC AAG GA-3′
*Lpin1a* forward	5′-GGT CCC CCA GCC CCA GTC CTT-3′
*Lpin1a* reverse	5′-GCA GCC TGT GGC AAT TCA-3′
*Srsf10* forward	5′-GTC CCA CTT GAT TTC TAC ACT CG-3′
*Srsf10* reverse	5′-TTC TCC TTT CAT AAC TCC GGC T-3′
*Pgc1a* forward	5′-TGA GGA CTG CTA GCA AGT TTG-3′
*Pgc1a* reverse	5′-AGT GAC CAA TCA GAA ATA ATA TCC AAT C-3′
*Lpin1* forward	5′-TCC CAG TTC GGA CAG AGA AT-3′
*Lpin1* reverse	5′-GCC AGA GCA TTT CCA GGT TA-3′
*Lpin1b* forward	5′-CAG CCT GGT AGA TTG CCA GA-3′
*Lpin1b* reverse	5′-GCA GCC TGT GGC AAT TCA-3′
*Pparg* forward	5′-CCA GAG CAT GGT GCC TTC GCT-3′
*Pparg* reverse	5′-CAG CAA CCA TTG GGT CAG CTC-3′
*Actb* forward	5′-GGC ACC ACA CCT TCT ACA ATG-3′
*Actb* reverse	5′-GGG GTG TTG AAG GTC TCA AAC-3′
*PRDM16* forward	5′-GAT GGG AGA TGCT GAC GGAT-3′
*PRDM16* reverse	5′-TGA TCT GAC ACA TGG CGA GG-3′
*Cidea* forward	5′-GTG TTA AGG AAT CTG CTG AG-3′
*Cidea* reverse	5′-CTA TAA CAG AGA GCA GGG TC-3′

**Figure A1:**
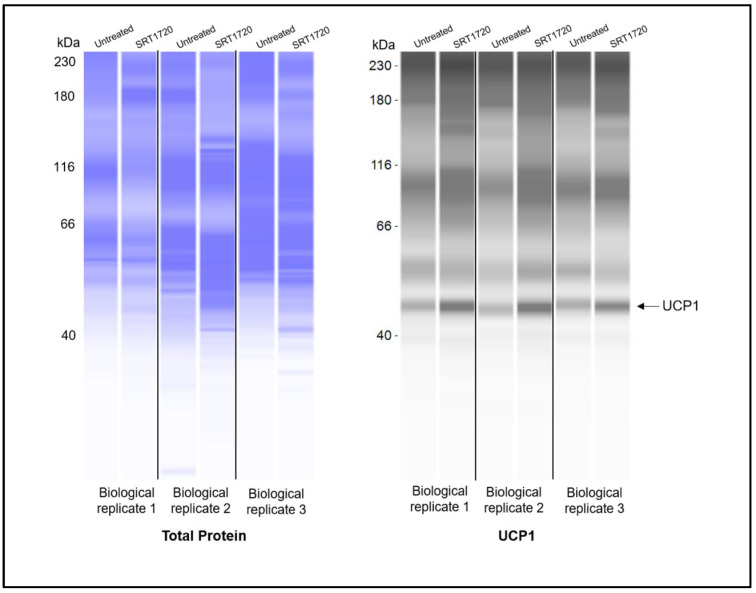
Replicates for UCP1 protein detection using Simple Western blot. UCP1 was consistently detected at ~47 Kda in all samples.

## Data Availability

The original contributions presented in this study are included in this article; further inquiries can be directed to the corresponding authors.
